# Supplementary material for: NOVAsort for error-free droplet microfluidics
Source: Nat Commun. 2024 Nov 1;15:9444. doi: 10.1038/s41467-024-52932-z (PMC11530522; doi:10.1038/s41467-024-52932-z)
Supplement: Supplementary file 1 — Supplementary Information [file 41467_2024_52932_MOESM1_ESM.pdf]

Title

NOVAsort for error-free droplet microfluidics

Authors

Han Zhang[1]†, Rohit Gupte[2]†, Yuwen Li[1], Can Huang[1], Adrian R. Guzman[1], Jeong Jae Han[3], Haemin Jung[1], Rushant Sabnis[4] Paul de Figueiredo[5,6,7] and Arum Han[1,2,4] *

Affiliations

[1] Department of Electrical and Computer Engineering, Texas A&M University, College Station, Texas 77843, USA

[2] Department of Biomedical Engineering, Texas A&M University, College Station, TX 77843, USA

[3] Department of Multidisciplinary Engineering, Texas A&M University, College Station, Texas 77843, United States

[4]Department of Chemical Engineering, Texas A&M University, College Station, TX 77843, USA

[5] Christopher S. Bond Life Science Center, University of Missouri, MO 65211USA

[6] Department of Molecular Microbiology and Immunology, School of Medicine, University of Missouri, MO 65212USA

[7] Department of Veterinary Pathobiology, University of Missouri, MO 65211, USA

†These authors contributed equally to this work: Han Zhang, Rohit Gupte

* This author (Arum Han, arum.han@ece.tamu.edu) is the corresponding author.

Supplementary figures

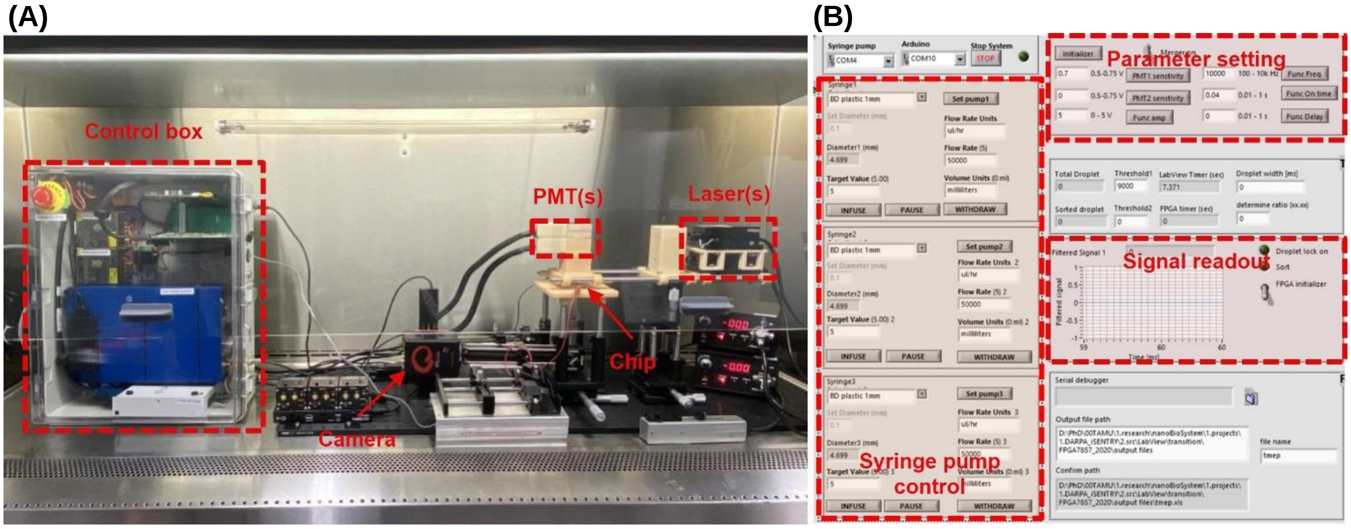

Supplementary figure 1. The fluorescence-activated droplet sorting platform. (A) Sorting station setup, including the electrical control sub-system (left) and the fluorescence detection sub-system (right). **(B)** LabVIEW^{TM} software user interface used for fluorescence-activated droplet sorting.

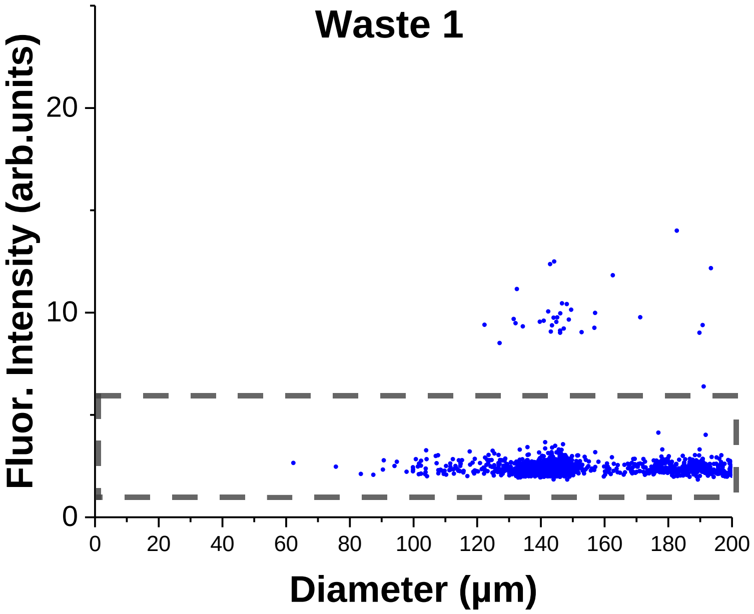

Supplementary figure 2. Performance of the NOVAsort using polydisperse droplet library. Droplets collected from waste 1 outlet of the NOVAsort.

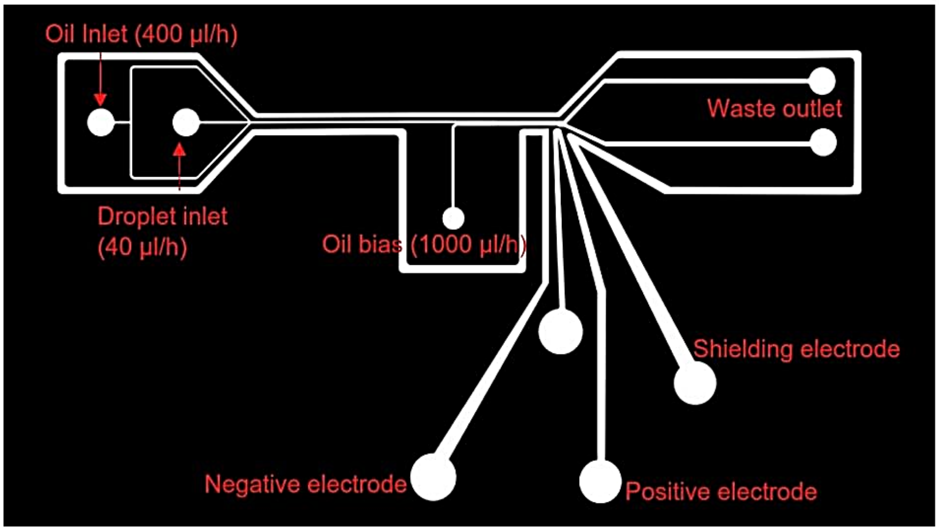

Supplementary figure 3. Design of the conventional linear sorter and flow rates of linear sorter used in the results shown in Figure 3B-D.

.

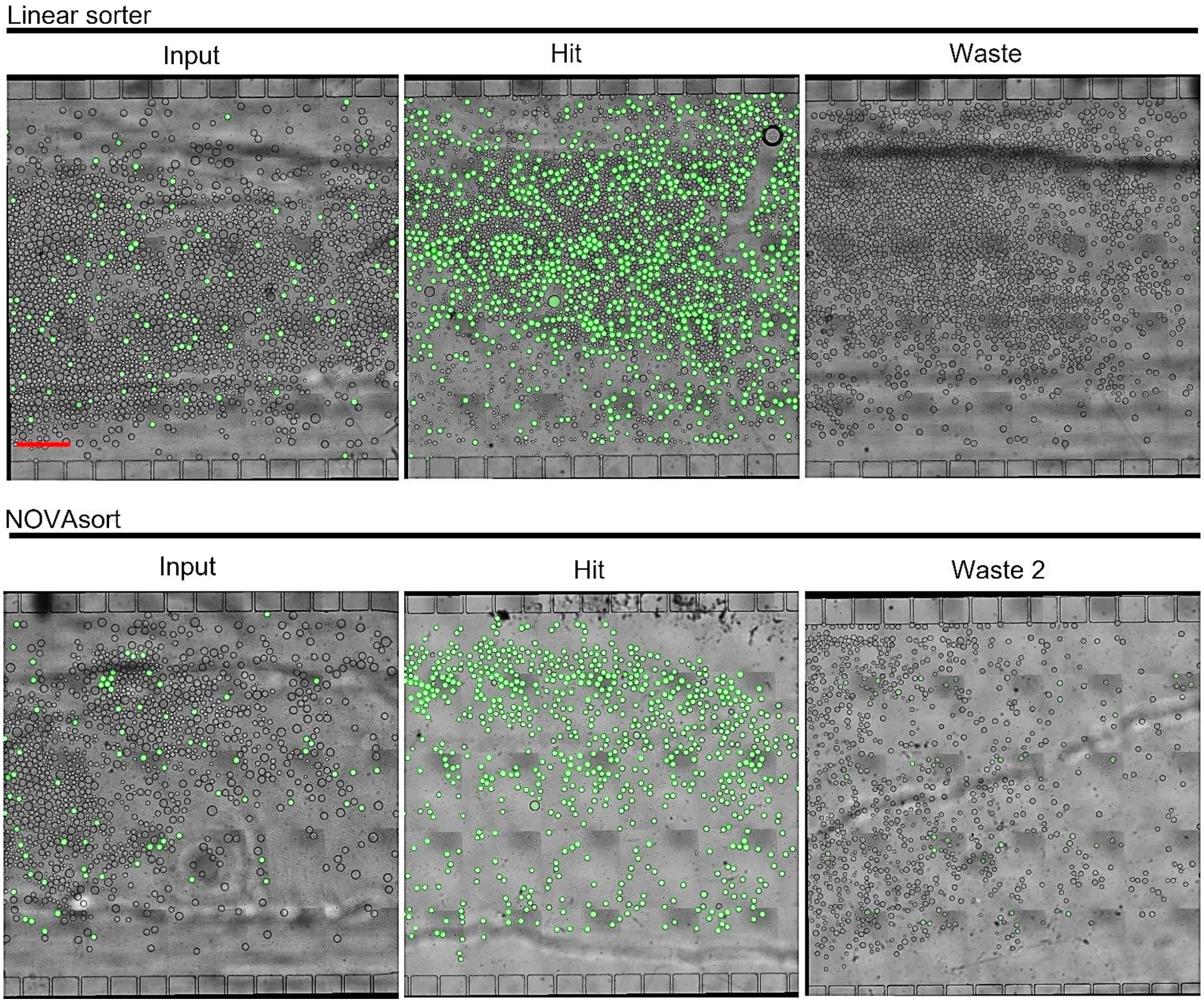

Supplementary figure 4. Performance comparison of NOVAsort against the linear sorter using a monodisperse droplet library (scale bar = 1 mm). Representative raw images for three separate experiments.

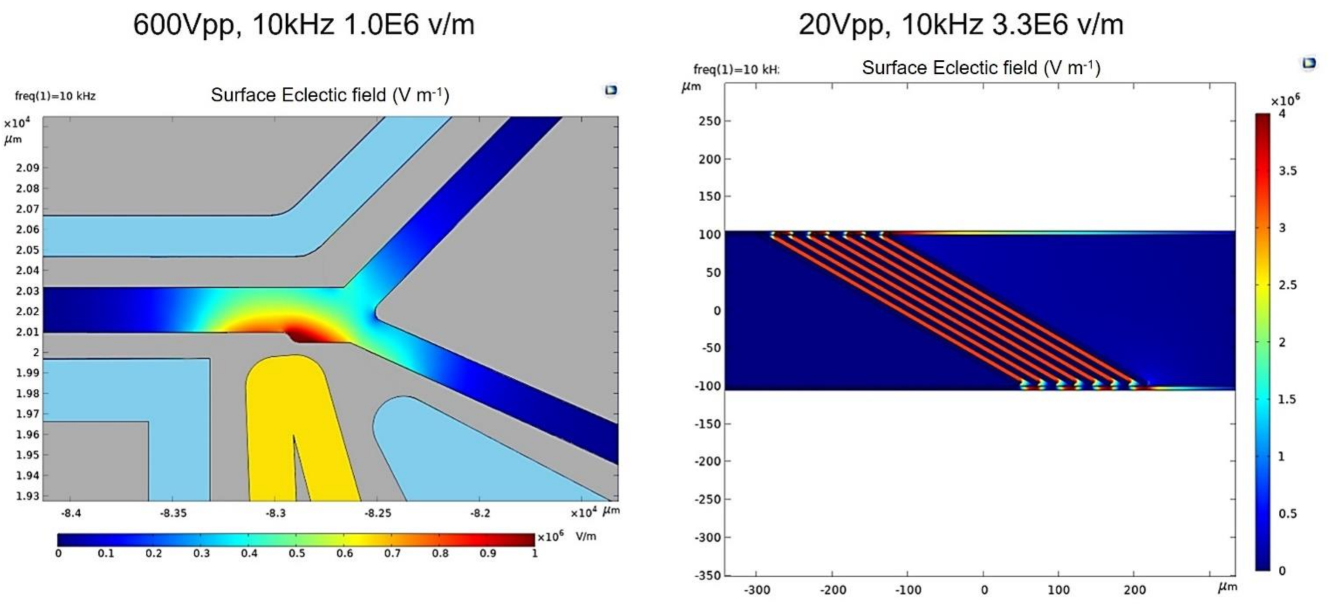

Supplementary figure 5. Finite element analysis of the electrical field generated from 3D liquid metal electrodes and IDEs. Top-view comparison of the simulated electrical field generated by the 3D liquid metal electrode of the conventional linear sorter (left) and by the surface IDEs used in NOVAsort (right).

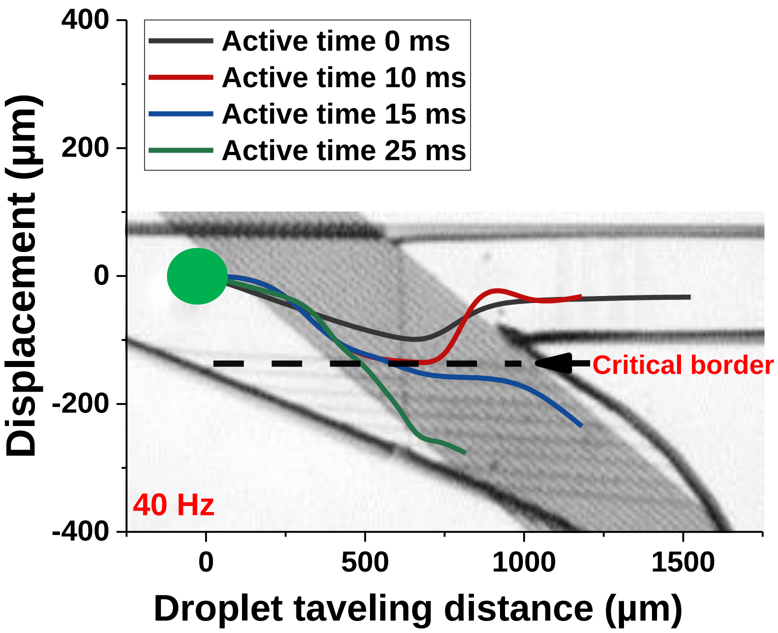

Supplementary figure 6. Sorting trajectories of droplets under different electrode active times at a throughput of 40 Hz. The position of the critical border is determined by the flow rate ratio between the hit outlet and the waste 2 outlet

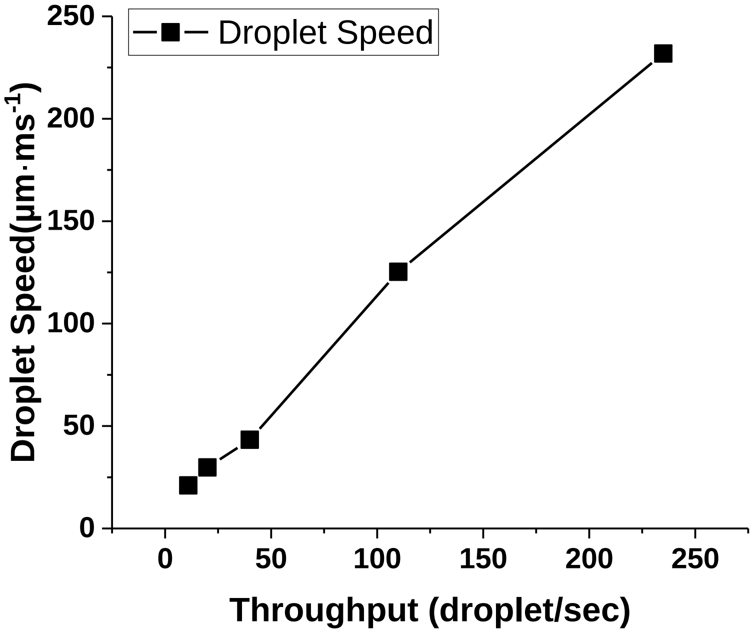

Supplementary figure 7. Calculated droplet flow speed within the sorting junction at different droplet throughputs for size of 120 µm.

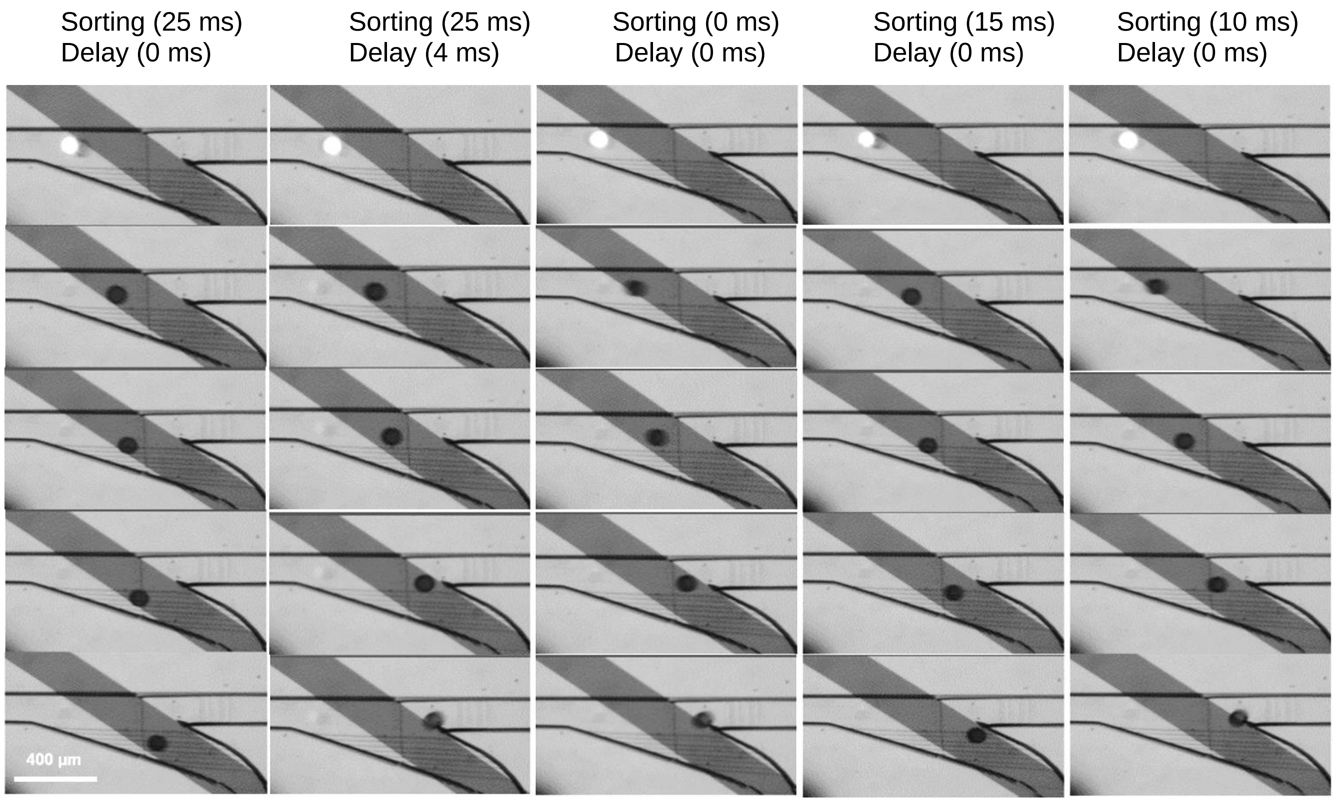

Supplementary figure 8. Frame-by-frame micrographs of NOVAsort operating under different sorting time and sorting delay times applied.

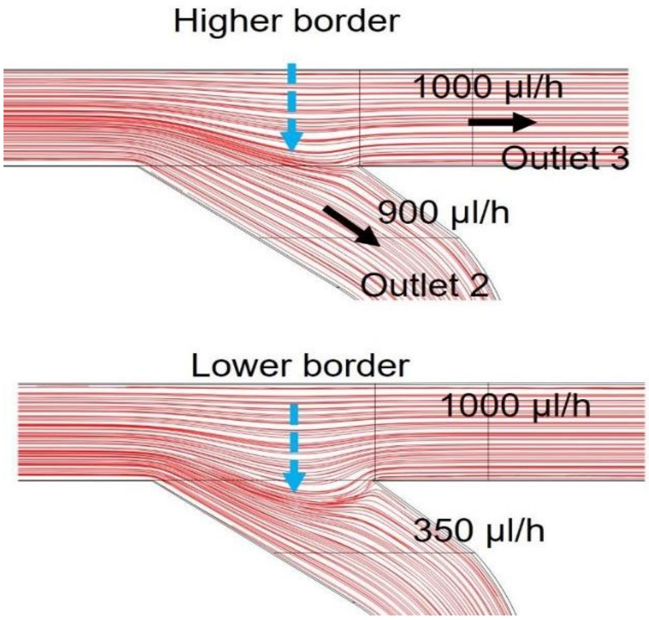

Supplementary figure 9. Finite element simulation showing the change in the critical borderline under different flow rate conditions. As the flow rate to outlet 2 decreases, the critical borderline shifts downward.

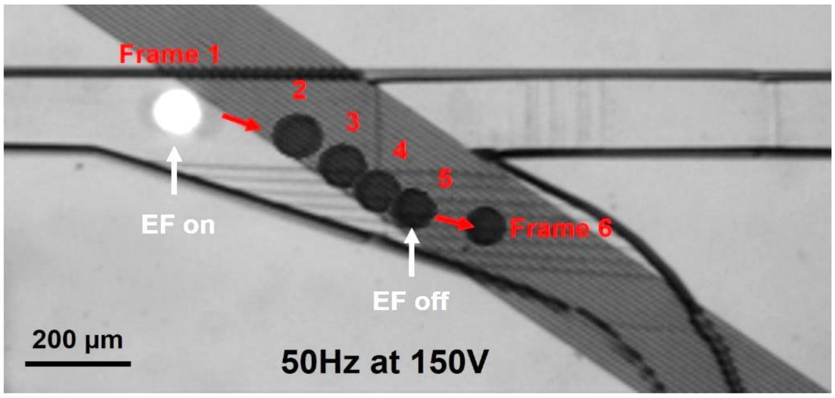

Supplementary figure 10. Droplet trajectory of NOVAsort when pulling a color dye-including droplet (120 µm in diameter). The surface IDE-generated DEP force allows the droplet to follow the path of the IDEs before it is released when the sorting channel moves away from the IDEs.

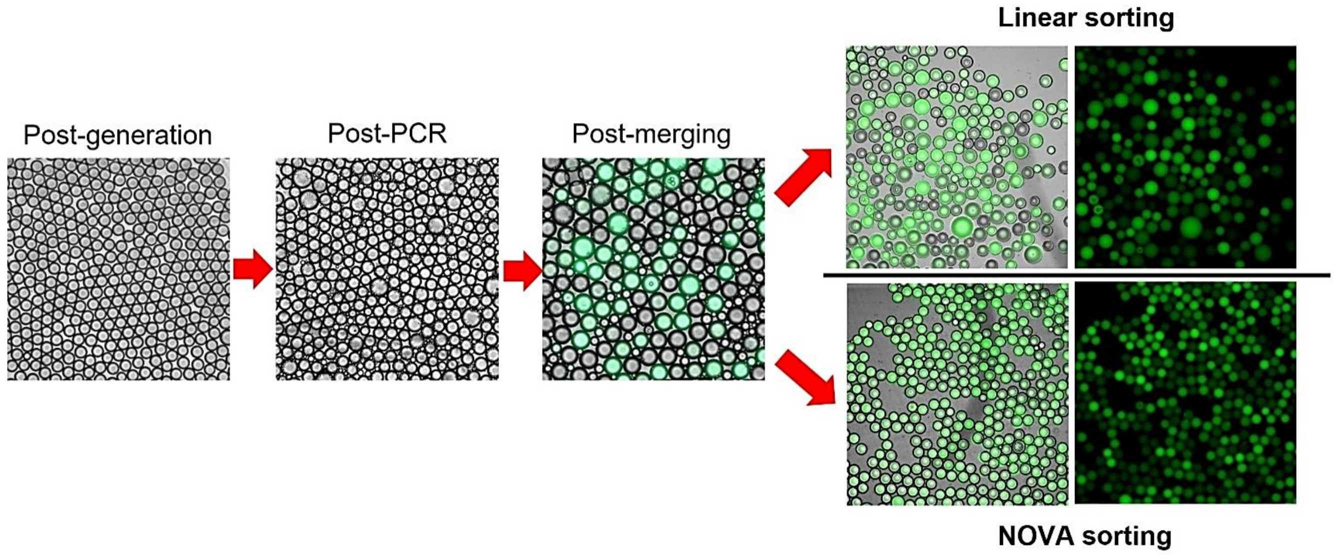

Supplementary figure 11. Sample images for statistical analysis during the various stages of the droplet IVTT assay workflow.

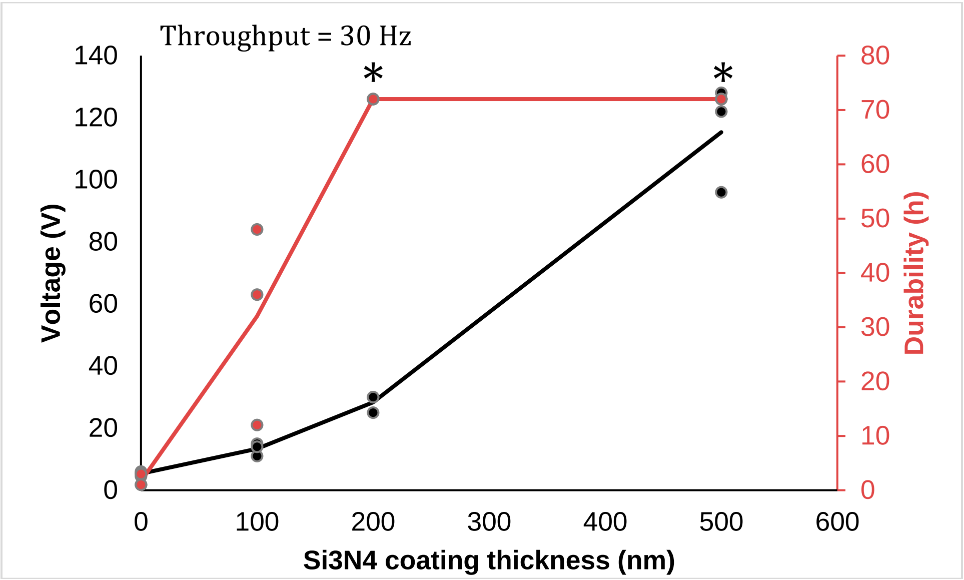

Supplementary figure 12. Correlation of Si_3N_4 coating thickness versus minimum operational voltage as well as versus durability of the electrodes (* indicates intact device after 72 h test). N = 3, indicates three separate experiments.

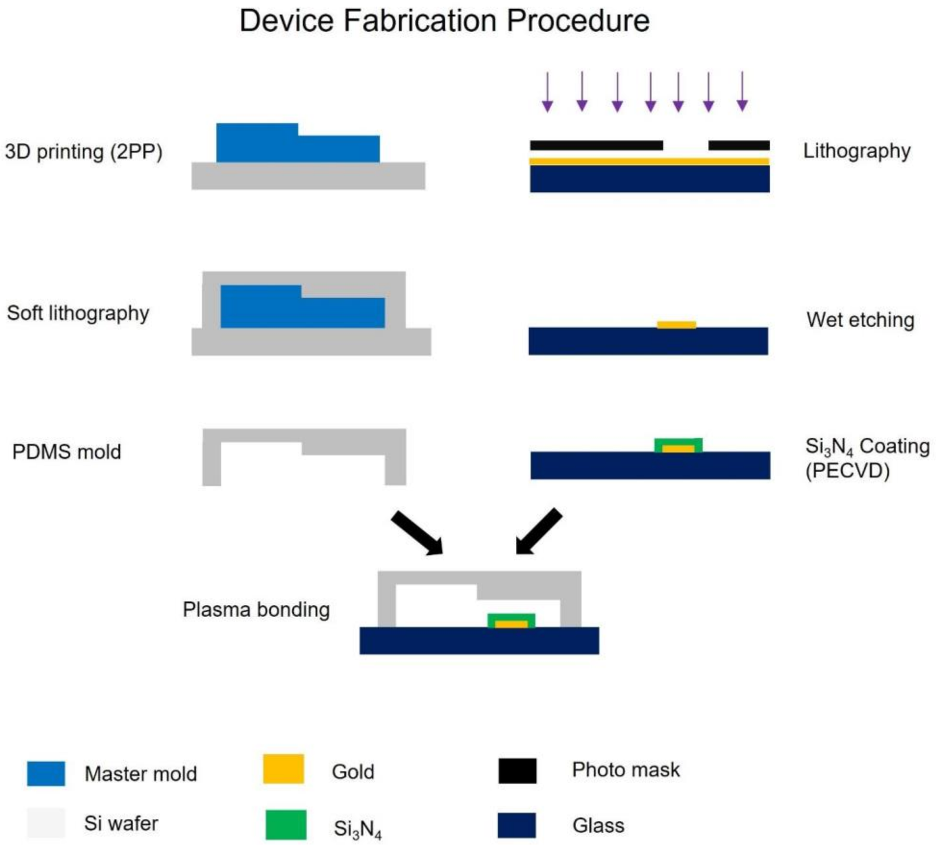

Supplementary figure 13. Fabrication steps of the NOVAsort device. The device is fabricated with 2-photon polymerization as wells as conventional lithography and metal deposition techniques.

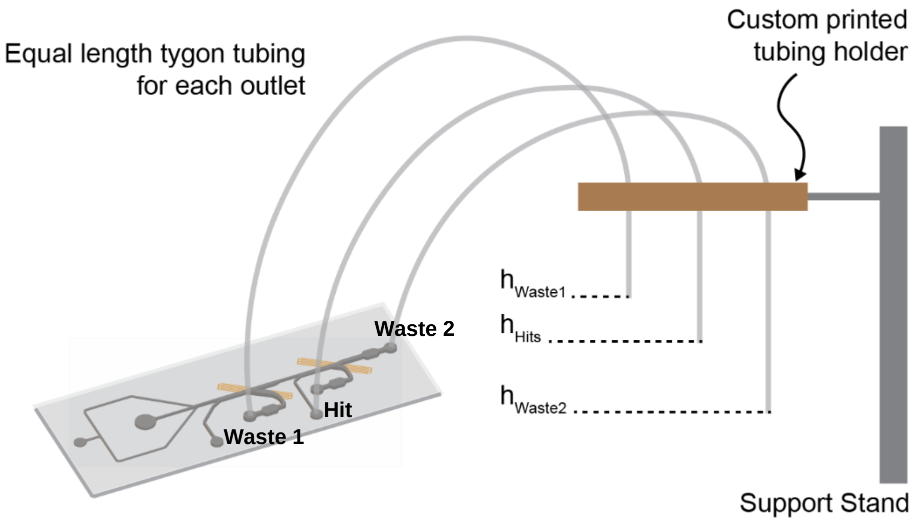

Supplementary figure 14. NOVAsort system hydrodynamic balancing setup. The waste 1 outlet is in general higher than the outlets of hits and waste 2.

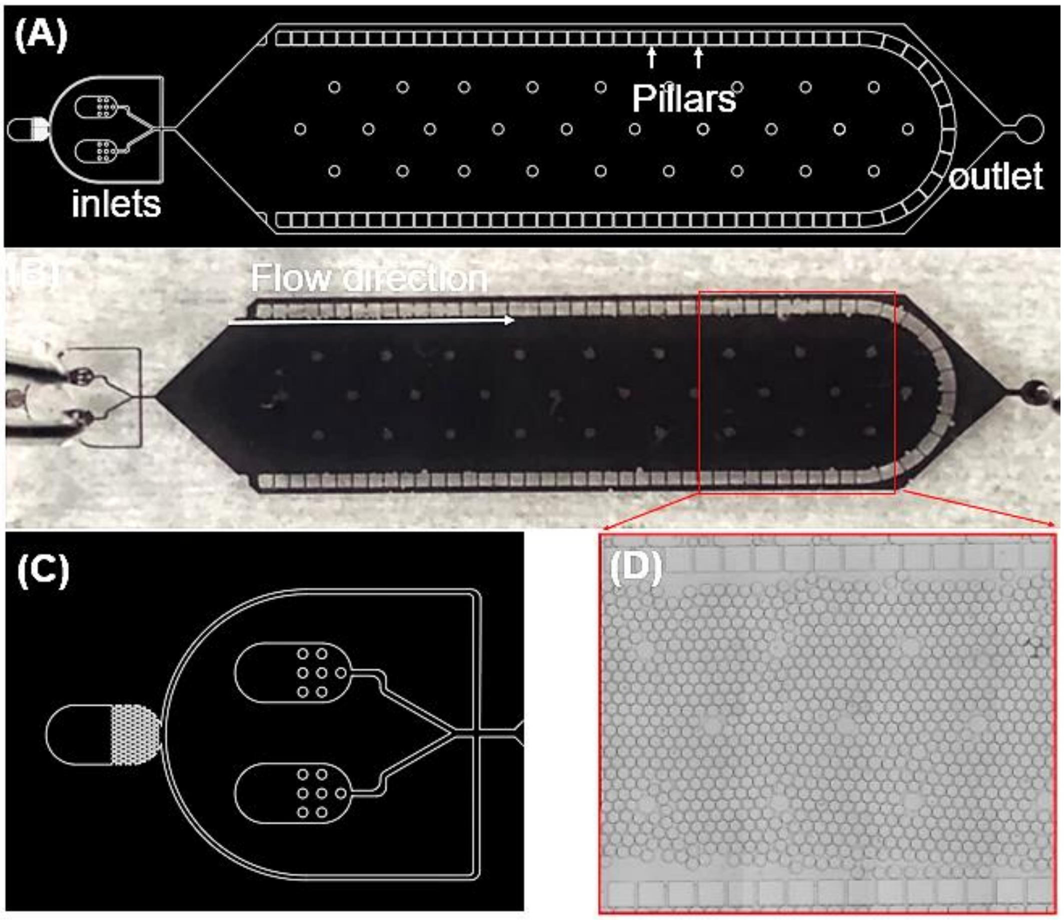

Supplementary figure 15. **The microfluidic basket chamber used to hold collected droplets.** (A) Design of the basket chamber. (B) Microfabricated chamber filled with black color dye for visualization. (C) Three inlets of the basket chamber can also be used for droplet generation. (D) Droplets are held in the chamber by the micropillar array.

Supplementary methods

Simulation and calculation of electrical field and fluidic profile in the droplet sorting region

COMSOL Multiphysics 6.1 (COMSOL Inc., Palo Alto, CA, USA) was used for the electric field simulation. The simulation was carried out across the cross-sectional view where the electrodes were either at the bottom of the channel (NOVAsort) or on both sides of the channel (conventional linear sorter). The channel height was set to 100 µm. The frequencies were fixed at 10 kHz, while the applied voltage was set as 600 V_{pp} for the linear sorter and 20 V_{pp} for the NOVAsort. A direct comparison of the electric field intensities at the middle height of the channel (50 µm, which is around the center of the floating droplets) showed that the electric field intensity of the linear sorter electrode was ~82x higher (Figure 4 E and F) than that of the NOVAsort surface IDEs, indicating a much greater chance of unwanted droplet merging during operation.

For the fluidic flow profile simulation, three assumptions were made to mimic the actual flow condition: (1) the fluid is Newtonian, (2) no-slip boundary condition, and (3) the flow within the channel is incompressible. The 3D models of the structures were created initially in AutoCAD 2020 (Autodesk Inc., San Rafael, CA, USA) and then imported into COMSOL Multiphysics. The simulation was performed using the physical interface laminar flow (spf) under a stationary study model. The material was set to Novac® oil with a density of 1614 $kg \cdot m^{-3}$ and a dynamic viscosity of 0.00125 Pa·s at 25°C.

Microfabrication of the vertically (z-direction) sloped droplet transition junction

Two-photon photolithography, also commonly termed two-photon polymerization (2PP), is a relatively new form of lithography that overcomes the limitations of conventional 2-dimensional microstructure fabrication methods. For the vertically sloped microstructure presented in this work, a 3D drawing of the structure was made using SolidWorks 2021 (Dassault Systems SolidWorks Corp., MA) to replicate the exact footprint of the sloped droplet

transition junction. The final SolidWorks file was then exported as an STL file and loaded to the DeScribe software (Nanosribe GmbH software, Germany) to prepare the STL file for path and job recognition. The prepared file was then uploaded to a two-photon polymerization tool, the Nanoscribe Photonics Professional GT2 (Nanoscribe GmbH, Germany), and the microstructure was fabricated using a negative photoresist (IP-S, Nanoscribe GmbH, Germany) at power scaling of 1.0, tetrahedron inner scaffold, base scan speed of 50,000, base laser power of 60%, shell/scaffold scan speed of 100,000, and shell/scaffold laser power intensity of 70%. All designs were fabricated on 4-inch silicon wafers. Following the printing, the wafers were removed from the tool and developed in propylene glycol monomethyl ether acetate (PGMEA, Millipore Sigma, MA) for 6 min. Following the PGMEA development process, the wafers containing the microstructures were placed in 99% proof isopropyl alcohol (IPA, VWR, PA) for 10 min to achieve fine development. Following the IPA development, the microstructures were dried with nitrogen gas and inspected under a microscope for quality assurance. This microstructure becomes the master mold for the subsequent soft lithography replication step.

Balancing the NOVAsort outlets

Three pieces of Tygon® tubing (Inner diameter = 250 µm) were cut to the same length (~36 mm. Each tubing was inserted into its outlet and hung from a custom 3D-printed tubing holder at the same height. All syringe pumps were turned on and infusion was started. Once stabilized (~120 s), section 2 (Figure 2a) was balanced by adjusting h_{waste1} (Supplementary figure 14) until the droplets hit just above the channel bifurcation region (elevating the tubing pushes the droplets away from waste1, lowering it brings them closer). The Bias oil 1 flow rate can be adjusted for fine control of droplet trajectories (a higher flow rate pushes the droplets away from waste 1, lowering the flow rate brings them closer). When optimally balanced and with the IDEs inactive, only carrier oil but no droplets flow out from waste 1. The first IDE was then activated to pull out the large droplets through waste 1. Next, section 4 (Figure 2a) was balanced by adjusting h_{hit} and h_{waste2} (Supplementary figure 14) until the droplets hit just above the channel bifurcation region. Care was taken to ensure the average of h_{hits} and h_{waste2} remains the same. For example, when h_{hit} was lowered, h_{waste2} was elevated by the same amount (elevating h_{hit} pushes the droplets away from the hits outlet, lowering it brings them closer). The bias oil 2 flow rate can be adjusted for fine control of the droplet trajectories (a higher flow rate pushes the droplets away from the hit outlet, and a lower flow rate brings them closer. This ensured that the flow at section 2 was not affected by the balancing of section 4. The second IDE was then fluorescent-activated to begin sorting.

Supplementary references

[1] H. Zhang *et al.*, "FIDELITY: A quality control system for droplet microfluidics," vol. 8, no. 27, p. eabc9108, 2022.
