# Supplementary material for: NOVAsort for error-free droplet microfluidics
Source: Nat Commun. 2024 Nov 1;15:9444. doi: 10.1038/s41467-024-52932-z (PMC11530522; doi:10.1038/s41467-024-52932-z)
Supplement: Supplementary file 3 — Description of Additional Supplementary Files [file 41467_2024_52932_MOESM3_ESM.pdf]

Title: Supplementary Movie 1
Description: Removal of oversized droplets from section 2

Title: Supplementary Movie 2
Description: Proof-of-cncept of NOVAsort using 6 different droplets

Title: Supplementary Movie 3
Description: Sorting (NOVAsort) using extreme polydisperse input library and at various droplet-to-droplet spacing

Title: Supplementary Movie 4
Description: Sorting (Linear and NOVAsort) using 5% and 95% hit droplets

Title: Supplementary Movie 5
Description: Failure modes of conventional linear sorter: unwanted merging and pulling multiple droplets

Title: Supplementary Movie 6
Description: NOVAsort overcomes the conventional failure modes: unwanted merging and pulling multiple droplets

Title: Supplementary Movie 7
Description: Failure modes of conventional linear sorter: droplet splitting

Title: Supplementary Movie 8
Description: NOVAsort overcomes the conventional failure modes: droplet splitting

Title: Supplementary Movie 9
Description: Failure mode of NOVAsort
